# Supplementary figures and images for: Static load test and bearing capacity analysis of broken line pretensioned prestressed concrete I-beam
Source: PLoS One. 2025 Aug 1;20(8):e0328562. doi: 10.1371/journal.pone.0328562 (PMC12316265; doi:10.1371/journal.pone.0328562)

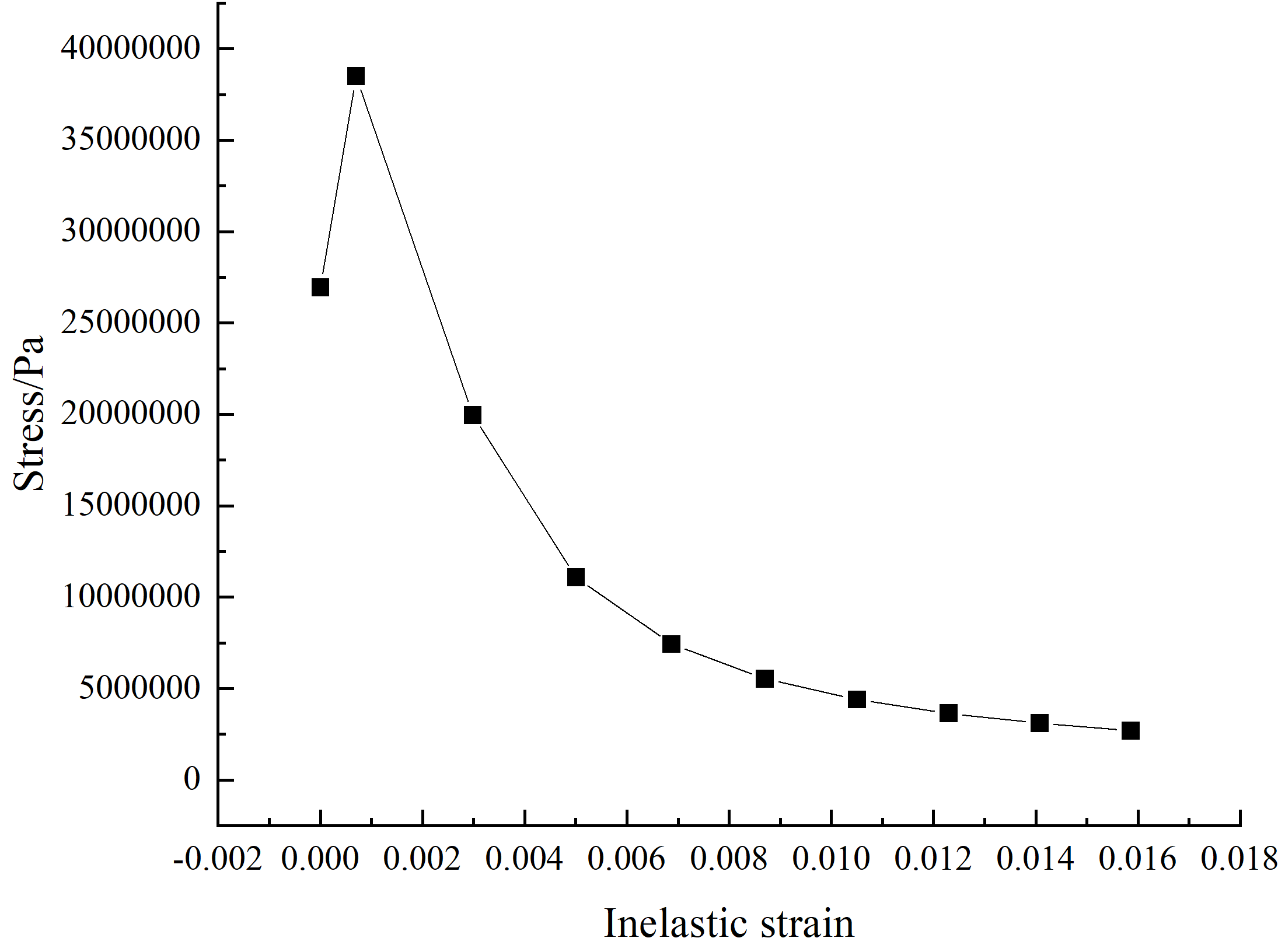

Supplement: S1 File — (ZIP) [file pone.0328562.s001.zip › Mechanical behavior of concrete/Compressive behavior of concrete(yield stage).png]

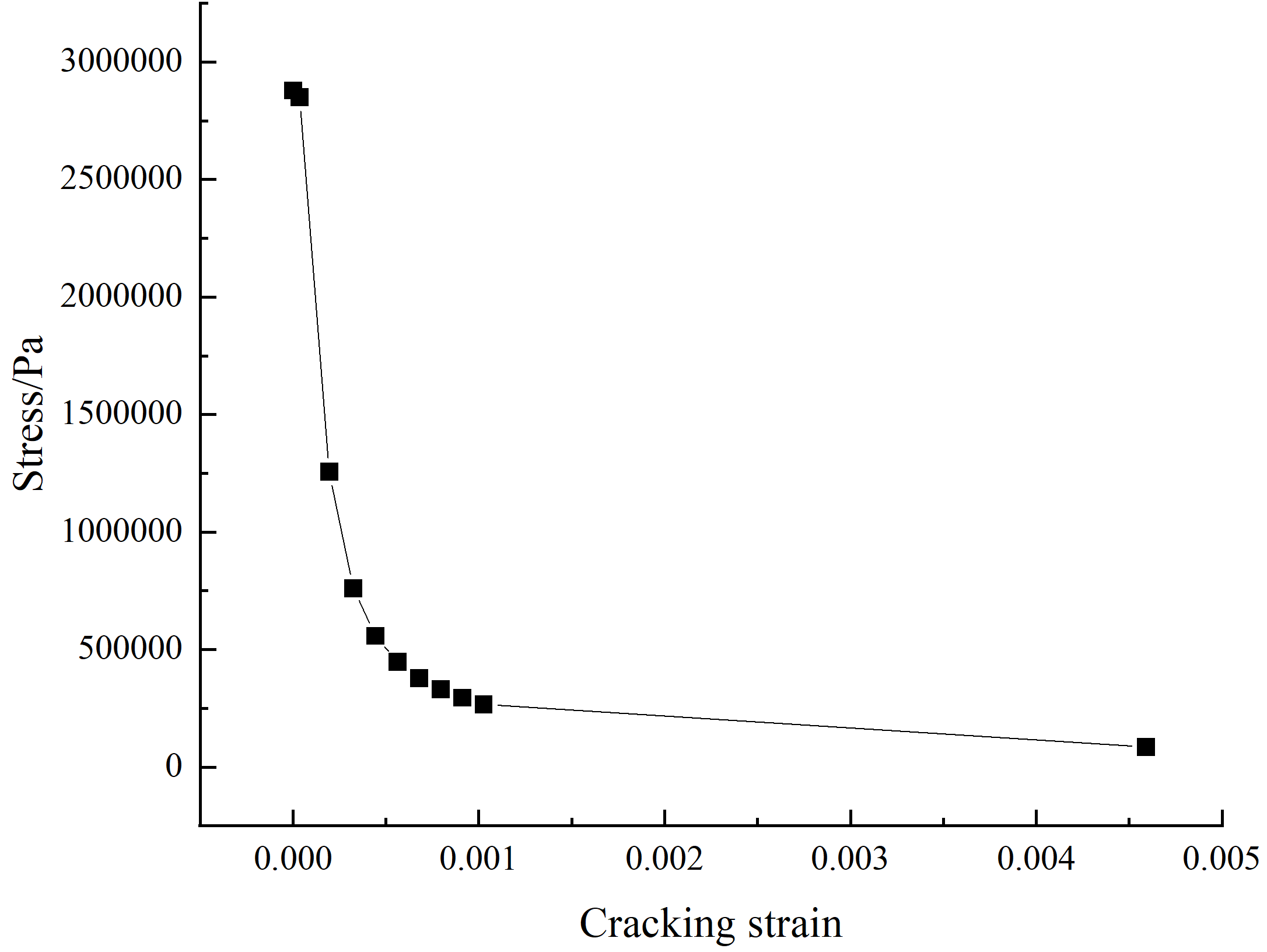

Supplement: S1 File — (ZIP) [file pone.0328562.s001.zip › Mechanical behavior of concrete/Tensile behavior of concrete(yield stage).png]
