# Supplementary material for: Static load test and bearing capacity analysis of broken line pretensioned prestressed concrete I-beam
Source: PLoS One. 2025 Aug 1;20(8):e0328562. doi: 10.1371/journal.pone.0328562 (PMC12316265; doi:10.1371/journal.pone.0328562)
Supplement: Attachment — Submitted filename: report.docx [file pone.0328562.s002.docx]

To improve and revise the paper titled "Static load test and bearing capacity analysis of broken line pretensioned prestressed concrete I-beam" here are some constructive suggestions:

- Clearly articulate the novelty of this study in the introduction. While the paper highlights that few studies exist on large-span broken line pretensioned I-beams, it should more explicitly state what distinguishes this research from previous work.
- Emphasize why the 30 m span and the chosen broken line tendon arrangement are practically significant.
- Reorganize the introduction to follow a clear logical flow: (i) background and existing challenges in prestressed beam design, (ii) shortcomings of linear tendon configurations, (iii) advantages of broken line configurations, (iv) the novelty and scope of the current study.
- Provide a clearer description of boundary conditions and simplifications used in the FE model (e.g., support type, contact assumptions).
- Elaborate on the validation process used to confirm the model accuracy, especially regarding the cooling method for prestress simulation.
- Specify mesh size, element quality, and convergence criteria used in ABAQUS for numerical simulations.
- The comparison between the two-fold and four-fold tendon arrangement methods could be enhanced by quantitative stiffness comparisons (e.g., change in slope of load-displacement curves).
- While some references are included, the literature review could be expanded to examine recent studies, such as:
- Rapid Analysis of CFRP-Reinforced Concrete Structures Using Artificial Neural Networks.
- Prediction of load-bearing capacity of RC Columns (CWA) using Artificial Neural Networks (ANN) trained on a Hybrid Experimental Database HEXP.
- Least squares collocation method in Moho depth determination in Iran using gravity gradient data.
