# Supplementary material for: Static load test and bearing capacity analysis of broken line pretensioned prestressed concrete I-beam
Source: PLoS One. 2025 Aug 1;20(8):e0328562. doi: 10.1371/journal.pone.0328562 (PMC12316265; doi:10.1371/journal.pone.0328562)
Supplement: Attachment — Submitted filename: Response to Reviewers.docx [file pone.0328562.s004.docx]

**Dear Reviewers:**

Thank you very much for the reviewers' valuable comments on the article, which are very helpful for us to improve the paper. There are indeed some problems in the first draft of the paper, and some parts of the presentation are not clear and precise enough, and the arguments are not sufficient. We have revised the paper carefully with the comments of the experts, and responded to the questions raised by the reviewers one by one, which is indicated in blue font in the corresponding position in the revised version of the paper, so that it is easy for you and the reviewers to review the paper further.

**To reviewer 1:**

**1. The model assumes perfect bond with no slip, which I think is inaccurate for draped or bent tendons in your case.**

Response to comment:

Thank you for the valuable comment, we fully agree with your insight that bond-slip effects in draped/bent tendons may affect the analysis results.

In the first draft, the “embeded” function is used to simulate the interaction between concrete and prestressed tendons. This function does not consider the bond-slip effect between concrete and prestressed tendons, and this simulation method is not accurate enough.

To study the bond-slip behavior between concrete and prestressed tendons, a bond-slip interface layer with a thickness of 0.1 mm is set between concrete and prestressed tendons in the revised manuscript. The interface layer is discretized by three-dimensional eight-node cohesive element (COH3D8), and the user subroutine developed by the Professor Fang is used to realize the nonlinear bond-slip constitutive relationship under external load. This method can reflect the degradation of interface stiffness and energy dissipation, and the rationality of the model has been obtained.

We have supplemented the model description in Section 3, and adjusted the simulation data in Section 4 and Section 5. The reference about user subroutine is as follows:

*[1] Fang Z H, Zhen Y, Li X P. Steel Hysteretic Model of Reinforced Concrete Structures. Engineering Journal of Wuhan University. 2018; 51(07): 613-619. doi: 10.14188/j.1671-8844.2018-07-008.*

**2. The simulation doesn't capturing the** **final failure modes, which might also be interesting to know.**

Response to comment:

Thank you for the valuable comment. In Section 5.1.2, the criterion of ultimate bearing capacity of the I-beam is defined:

(1) The maximum compressive strain of concrete reaches;

(2) The prestressed tendon reaches yield state;

(3) The deflection of the mid-span section exceeds the limit value.

In the numerical simulation, when the concentrated load reaches 1910 kN, the individual prestressed tendons at the bottom of the I-beam enter the yield state. At this time, the maximum compressive strain of the concrete is less than ultimate strain and the deflection of the mid-span section does not exceed the limit value. Therefore, the prestressed tendons play a controlling role in the ultimate bearing capacity of the I-beam. When the concentrated load reaches 1921 kN, the I-beam cannot continue to bear the load.

In the first draft, we only give the damage situation of the I-beam when individual prestressed tendons enter the failure state in Figure 15. In the revised manuscript, we have added the final failure mode. We have also added the relevant description, which will help the readers understand the whole process of the I-beams from loading to failure.

(a)

(b)

(c)

Figure 15. Damage situation of I-beams at different stages (unit: cm): (a) Local concrete at the bottom cracks; (b) Individual prestressed tendons enter the yield state; (c) Peak load state.

**3. More details should be included for the FEM model setup. For instance the element size, key assumption, etc.**

Response to comment:

Thank you for the valuable comment. In the first draft, we did not provide a detailed description of the finite element model, and the assumptions of numerical simulation. We have made corresponding supplements in the revised manuscript.

(1) Element size

In general, the denser the grid is, the closer the calculated results of the model are to the true value. But it will lead to a rapid increase in the amount of calculation and reduce the computational efficiency. Considering the calculation accuracy and calculation efficiency, the element size of concrete, prestressed tendons and ordinary steel bars of I-beam is determined to be about 10 cm. Since the loading pad in the mid-span is not the main research focus, the element size is determined to be about 50 cm.

(2) Contact assumption

To study the bond-slip behavior between concrete and prestressed tendons, a bond-slip interface layer is set between concrete and prestressed tendons. The interface layer is discretized by three-dimensional eight-node cohesive element (COH3D8), and the user subroutine is used to realize the nonlinear bond-slip constitutive relationship under external load. The ordinary steel bars are embedded into concrete through the “embedded” function of ABAQUS to achieve collaborative deformation, which does not consider the bond-slip behavior between ordinary steel bars and concrete.

(3) Boundary condition

In the field test, the test beam was in an approximately simply supported state. In the numerical simulation, the ideal simple support constraint is applied to the I-beam model. One end of the beam constrains the linear displacement in three directions, and the other end constrains the linear displacement in two directions except the axis direction.

The additional explanations are shown in Section 3 in the revised manuscript.

**4. It would be valuable for the authors to compare or discuss the potential of physics informed neural network (PINN)-based methods as an alternative or complementary tool to FEM-based modeling**

Response to comment:

Thank you for the valuable comment. PINN is an effective machine learning method that integrates physical laws into deep learning models. It is mainly used to solve differential equations, inverse problems and multi-physics coupling analysis. In the field of bridge engineering, PINN can be used for intelligent design, intelligent construction and intelligent detection.

In this paper, the research focus is on the ultimate bearing capacity of I-beam and the preliminary optimization of the arrangement of prestressed tendons. In the future research, we will use the PINN method to carry out intelligent detection of bridge structures and propose an optimized design scheme for the I-beam.

In the revised manuscript, we have improved the conclusions and proposed the prospects for future research.

**5. Following on previous comments, I suggest to include the recent work of PINN for structural analysis. For instance "Physics-informed neural networks for system identification of structural systems with a multiphysics damping model" and "Physics-informed neural network for nonlinear structural system identification".**

Response to comment:

Thank you for the valuable comment. PINN is an effective machine learning method in the field of civil engineering. We believe that adding the recent work of PINN for structural analysis in the introduction can make the literature review more comprehensive.

In the revised manuscript, we have added the literatures mentioned by the reviewers to the introduction, and the references are as follows:

*[1]Liu T, Meidani H. Physics-informed neural networks for system identification of structural systems with a multiphysics damping model. Journal of Engineering Mechanics. 2023; 149(10): 04023079. doi:10.1061/JENMDT.EMENG-7060.*

*[2]Liu T, Meidani H, editors. Physics-Informed Neural Network for Nonlinear Structural System Identification. 14th International Workshop on Structural Health Monitoring: Designing SHM for Sustainability, Maintainability, and Reliability, IWSHM 2023, September 12, 2023 - September 14, 2023; 2023; Stanford, CA, United states: DEStech Publications. doi: 10.12783/shm2023/37077.*

**6. I suggest to separate the first introduction into two sections (introduction and related work.**

Response to comment:

Thank you for the valuable comment. In the first draft, our description of introduction is not clear enough. In the revised manuscript, we have divided introduction into two parts:

(1) Research background

In this part, we introduced the broken line pretensioned construction technology and its application in bridge engineering.

(2) Research status

In this part, we introduced the related work of experts and scholars in theoretical analysis, experimental test and numerical simulation. After summarizing the research status, the research content of this paper was introduced.

**To review 2:**

**1. Clearly articulate the novelty of this study in the introduction. While the paper highlights that few studies exist on large-span broken line pretensioned I-beams, it should more explicitly state what distinguishes this research from previous work.**

Response to comment:

Thank you for the valuable comment. The novelty of this study and its differences from previous studies have been clearly stated in the introduction of the revised manuscript. The specific description is as follows.

Although there has been some research on the construction technology of the broken line pretensioned method, there are still some limitations in the related research:

(1) The experimental subjects are predominantly rectangular beams and T-beams, the research on I-beams with better section efficiency is insufficient.

(2) In the existing experimental research, the span of the beam is generally less than 20m, lacking systematic studies on the elastoplastic evolution and ultimate bearing capacity of large-span prestressed concrete structures.

(3) There is a lack of research on the optimal arrangement of prestressed tendons.

In the construction of bridge engineering, a standard design span of 30m can effectively balance structural economy and engineering rationality while meeting the traffic needs. It can not only avoid the problem of high pier density caused by small span, but also avoid the structural redundancy caused by large span, so as to reduce the cost of foundation construction and the waste of building materials. Therefore, the specialized research on 30m span I-beam has significant reference value for similar bridge projects.

For this question, we have made a supplementary explanation in Section 1.2 in the revised manuscript.

**2.** **Emphasize why the 30 m span and the chosen broken line tendon arrangement are practically significant.**

Response to comment:

Thank you for the valuable comment. Here is a supplementary explanation on the research value of 30m span beam and broken line tendon arrangement:

Similar to the previous question, a standard design span of 30m can effectively balance structural economy and engineering rationality while meeting the traffic needs. It can not only avoid the problem of high pier density caused by small span, but also avoid the structural redundancy caused by large span, so as to reduce the cost of foundation construction and the waste of building materials. The research on 30m span I-beam has engineering application value.

In the conventional pretensioning construction technique, the prestressed tendons are typically arranged in a linear configuration, which cannot accurately reflect the actual moment distribution curve of the structural components. The distribution of prestress on each section of the component and the corresponding external load effect fail to achieve the best matching state, especially at the end region of the beam. When the span of the beam is large, the disadvantage of linear arrangement of prestressed tendons will be more prominent. The broken line pretensioned construction technology can make the concrete near the beam end subject to pre-shear force, effectively control the generation of diagonal cracks at the beam end, enhance the section bending strength, reduce the use of materials, and improve the mechanical performance of the beam.

The related supplements have been added to Section 1 in the revised manuscript. We have also added relevant references:

*[1]Liu X F, Chen L J, Design of Pretensioned Reinforced Concrete Beam with Zigzag Reinforcement. Railway Standard Design.2004;(3):1-5. doi: 10.3969/i.issn.1004-2954.2004.03.001.*

*[2]Tong Q, Guo ZQ. A Design Practice of Pretensioned Prestressed Beams with Broken Line Tendons.Shanxi Architecture. 2010; 36(19): 332-333. doi: 10.3969/i.issn.1009-7767.2010.04.016.*

*[3]Li XM. Comparison on the Economyof medium and small beam bridge . ME. Thesis, Chang'an University. 2010. doi: 10.7666/d.y1729212.*

*[4]Zhai D. Analysis of the influence of economic indicators of viaduct based on Hangzhou Tongcheng Expressway Project. Project Management. 2018; (08): 16-49. doi: 10.15968/j.cnki.jsjl.2018.08.016.*

**3. Reorganize the introduction to follow a clear logical flow: (i) background and existing challenges in prestressed beam design, (ii) shortcomings of linear tendon configurations, (iii) advantages of broken line configurations, (iv) the novelty and scope of the current study.**

Response to comment:

Thank you for the valuable comment. In the first draft, the logic of the introduction is not clear enough. We have adjusted the introduction.

In Section 1.1 “Research background”, we introduce the following two parts:

(1) Background and challenges, as well as the disadvantages of linear arrangement of prestressed tendons.

(2) Advantages of the broken line pretensioned construction technology.

In Section 1.2 “Research status”, we introduce the following two parts:

(1) The related work of experts and scholars in theoretical analysis, experimental test and numerical simulation.

(2) The research content and novelty of this paper.

We have also added relevant references:

*[1]Amorn W, Tuan CY, Tadros MK. Curved, precast, pretensioned concrete I-girder bridges. PCI journal 2008: 48-66. doi: 10.15554/pcij.11012008.48.66.*

*[2]Oh BH, Kim ES, Choi Yc. Theoretical analysis of transfer lengths in pretensioned prestressed concrete members. Journal of engineering mechanics. 2006; 132(10): 1057-1066. doi:10.1061/(ASCE)0733-9399(2006)132:10(1057).*

*[3]Markovič M, Krauberger N, Saje M, Planinc I, Bratina S. Non-linear analysis of pre-tensioned concrete planar beams. Engineering Structures. 2013;46:279-93. doi: <https://doi.org/10.1016/j.engstruct.2012.08.004>.*

*[4]Liu X F, Chen L J, Design of Pretensioned Reinforced Concrete Beam with Zigzag Reinforcement. Railway Standard Design.2004;(3):1-5. doi: 10.3969/i.issn.1004-2954.2004.03.001.*

*[5]Tong Q, Guo ZQ. A Design Practice of Pretensioned Prestressed Beams with Broken Line Tendons.Shanxi Architecture. 2010; 36(19): 332-333. doi: 10.3969/i.issn.1009-7767.2010.04.016.*

*[6]Li XM. Comparison on the Economyof medium and small beam bridge . ME. Thesis, Chang'an University. 2010. doi: 10.7666/d.y1729212.*

*[7]Zhai D. Analysis of the influence of economic indicators of viaduct based on Hangzhou Tongcheng Expressway Project. Project Management. 2018; (08): 16-49. doi: 10.15968/j.cnki.jsjl.2018.08.016.*

**4. Provide a clearer description of boundary conditions and simplifications used in the FE model (e.g., support type, contact assumptions).**

Response to comment:

Thank you for the valuable comments. In the first draft, we did not provide a clear description of the finite element model. We have made corresponding supplements in the revised manuscript.

(1) Contact assumption

To study the bond-slip behavior between concrete and prestressed tendons, a bond-slip interface layer is set between concrete and prestressed tendons. The interface layer is discretized by three-dimensional eight-node cohesive element (COH3D8), and the user subroutine is used to realize the nonlinear bond-slip constitutive relationship under external load. The ordinary steel bars are embedded into concrete through the “embedded” function of ABAQUS to achieve collaborative deformation, which does not consider the bond-slip behavior between ordinary steel bars and concrete.

(2) Boundary condition

In the field test, the test beam was in an approximately simply supported state. In the numerical simulation, the ideal simple support constraint is applied to the I-beam model. One end of the beam constrains the linear displacement in three directions, and the other end constrains the linear displacement in two directions except the axis direction.

The additional explanations are shown in Section 3 in the revised manuscript.

**5. Elaborate on the validation process used to confirm the model accuracy, especially regarding the cooling method for prestress simulation.**

Response to comment:

Thank you for the valuable comment. In the first draft, we did not adequately describe the validation of model accuracy. In the revised manuscript, we have made the corresponding supplement, mainly in the following two aspects:

(1) Decreasing temperature method (cooling method)

The description of the cooling method is not accurate enough. In the revised manuscript, the cooling method has been changed to the decreasing temperature method.

In ABAQUS, the decreasing temperature method is a commonly used method to apply prestress. By reducing the temperature of the prestressed tendon unit, the shrinkage force generated by the shrinkage of the prestressed tendon is used to simulate the effect of the prestressed tendon on the concrete. The magnitude of the prestress can be controlled by the cooling value, which is calculated by the principle that the linear strain generated by the temperature is equal to the linear strain generated by the axial force. The calculation formula is as follows.

$$T=\frac{F_{T}}{\alpha\times E_{s}\times A_{s}}$$

where *T* is the temperature value, *F_T_* is the control tension of prestressed tendons, *E_s_* is the elastic modulus, *A_s_* is the cross-sectional area, and α is the linear expansion coefficient, which is set to 1.2×10^-5^.

The rationality of the decreasing temperature method has been verified by relevant scholars, and the relevant references are as follows. In this paper, the rationality of the numerical simulation method is also verified by the data comparison of the arch height of the I-beam.

We have also added relevant references:

*[1] Al-Ilani M, Temsah Y. Comparative study of modeling methods used to simulate initial stresses in prestressed beams towards manual analysis. MATEC Web of Conferences. 2019;281. doi: 10.1051/matecconf/201928101014.*

*[2] Lin SC., Song J, Gao S, Guo JP, Zhou YJ, Wang Y Q. Numerical simulation and field test studies on mechanical behavior of steel plate girder strengthened by external prestressed tendon. Structures. 2021; 33: 3188-3201. doi: <https://doi.org/10.1016/j.istruc.2021.06.048>.*

(2) Rationality of finite element model

In Section 4, the experimental data and simulation data are compared in terms of arch height, strain, displacement and neutral axis position. In Section 5, the results of numerical simulation and theoretical analysis are compared in terms of ultimate bearing capacity. The comparison results verify the rationality of the finite element model and the numerical simulation method.

The relevant modifications can be found in Section 3, Section 4 and Section 5 of the revised manuscript

**6. Specify mesh size, element quality, and convergence criteria used in ABAQUS for numerical simulations.**

Response to comment:

Thank you for the valuable comments. Here are some additional explanations for numerical simulations.

(1) Mesh size

In general, the denser the grid is, the closer the calculated results of the model are to the true value. But it will lead to a rapid increase in the amount of calculation and reduce the computational efficiency. Considering the calculation accuracy and calculation efficiency, the element size of concrete, prestressed tendons and ordinary steel bars of I-beam is determined to be about 10 cm. Since the loading pad in the mid-span is not the main research focus, the element size is determined to be about 50 cm.

(2) Element quality

In terms of element selection, the C3D8R element is used to simulate the concrete, B31 element is used to simulate the prestressed tendons, and the T3D2 element is used to simulate the ordinary steel bars. These elements are also commonly used in ABAQUS and have good simulation effects on the corresponding materials.

(3) Convergence problem

The elements selected in the finite element model are very regular, especially the three-dimensional eight-node reduced integral element is used to simulate the concrete. Through trial calculations, the most suitable mesh size is selected. By reasonably selecting the element type and mesh size, setting the incremental step and adjusting the contact convergence tolerance, the calculation convergence is ensured.

The relevant modifications can be found in Section 3 of the revised manuscript

**7. The comparison between the two-fold and four-fold tendon arrangement methods could be enhanced by quantitative stiffness comparisons (e.g., change in slope of load-displacement curves).**

Response to comment:

Thank you for the valuable comment. In Figure 17 of the first draft, we qualitatively compare the slopes of the two curves. In the revised draft, we supplement the quantitative comparison.

The slopes of the two curves are constantly changing in the yield stage, therefore, the slopes at a vertical displacement of 75mm and 100mm are selected for comparison.

(1) When the vertical displacement is 75 mm, the curve slope of four-fold point method K_4_ is 9.03kN/mm, which is 14.60% higher than the curve slope of two-fold point method K_2_ of 7.88kN/mm.

(2) When the vertical displacement is 100 mm, the curve slope of four-fold point method K_4_ is 6.57kN/mm, which is 8.96% higher than the curve slope of two-fold point method K_2_ of 6.03kN/mm.

The supplementary explanations can be found in Section 5.2 of the revised manuscript

**8. While some references are included, the literature review could be expanded to examine recent studies, such as:**

**“Rapid Analysis of CFRP-Reinforced Concrete Structures Using Artificial Neural Networks.”**

**“Prediction of load-bearing capacity of RC Columns (CWA) using Artificial Neural Networks (ANN) trained on a Hybrid Experimental Database HEXP.”**

**“Least squares collocation method in Moho depth determination in Iran using gravity gradient data.”**

Response to comment:

Thank you for the valuable comment. We believe that the literature mentioned by the reviewer can make the literature review in the introduction more comprehensive. We have added them to the references and introduced them in this paper.

The added references are as follows:

*[1]Heydarizadeh Shali H, Iapige De Gaetani C, Barzaghi R, Ramouz S, Safari A, Betti B, Abbasi Hafshejani Z. Least squares collocation method in Moho depth determination in Iran using gravity gradient data. Heliyon. 2024 Jan 23;10(3):e24596. doi: 10.1016/j.heliyon.2024.e24596. PMID: 38318048; PMCID: PMC10839806.*

*[2]Shakouri Mahmoudabadi N, Camp CV. Rapid Analysis of CFRP-Reinforced Concrete Structures Using Artificial Neural Networks. In: Ahmad A, Camp CV, editors. Advanced Optimization Applications in Engineering. Hershey, PA, USA: IGI Global Scientific Publishing; 2024. p. 60-96.*

*[3]Al-Sayegh AT, Mahmoudabadi NS, Shabbir F, Alkandari FJ, Saghir S, Ahmad A. Prediction of load-bearing capacity of RC columns (CWA) using Artificial Neural Networks (ANN) trained on a hybrid experimental database HEXP. Journal of Engineering Research. 2024. doi: <https://doi.org/10.1016/j.jer.2024.12.014>.*
